# Association of Auditory Interference and Ocular-Motor Response with Subconcussive Head Impacts in Adolescent Football Players

**DOI:** 10.1089/neur.2023.0125

**Published:** 2024-05-31

**Authors:** Zachary S. Bellini, Grace O. Recht, Taylor R. Zuidema, Kyle A. Kercher, Sage H. Sweeney, Jesse A. Steinfeldt, Keisuke Kawata

**Affiliations:** ^1^ Department of Kinesiology, Indiana University School of Public Health-Bloomington, Bloomington, Indiana, USA.; ^2^ Department of Neuroscience, Pomona College, Claremont, California, USA.; ^3^ Program in Neuroscience, The College of Arts and Sciences, Indiana University, Bloomington, Indiana, USA.; ^4^ Department of Counseling and Educational Psychology, School of Education, Indiana University, Bloomington, Indiana, USA.; ^5^ Department of Pediatrics, Indiana University School of Medicine, Indianapolis, Indiana, USA.

**Keywords:** traumatic brain injury, concussion, neuro-ophthalmologic function, ocular-motor, King–Devick, subconcussive head impacts

## Abstract

The aim of this study was to examine whether neuro-ophthalmological function, as assessed by the King–Devick test (KDT), alters during a high school football season and to explore the role of auditory interference on the sensitivity of KDT. During the 2021 and 2022 high school football seasons, football players’ neuro-ophthalmological function was assessed at five time points (preseason, three in-season, postseason), whereas control athletes were assessed at preseason and postseason. Two-hundred ten football players and 80 control athletes participated in the study. The year 1 cohort (*n* = 94 football, *n* = 10 control) was tested with a conventional KDT, whereas the year 2 cohort (*n* = 116 football, *n* = 70 control) was tested with KDT while listening to loud traffic sounds to induce auditory interference. There were improvements in KDT during a season among football players, regardless of conventional KDT (preseason 53.4 ± 9.3 vs. postseason 46.4 ± 8.5 sec; β = −1.7, SE = 0.12, *p* < 0.01) or KDT with auditory interference (preseason 52.3 ± 11.5 vs. postseason 45.1 ± 9.5 sec; β = −1.7, SE = 0.11, *p* < 0.001). The degree of improvement was similar between the tests, with no significant group-by-time interaction (β = −0.08, SE = 0.17, *p* = 0.65). The control athletes also improved KDT performance at a similar degree as the football cohorts in both KDT conditions. Our data suggest that KDT performance improves during a season, regardless of auditory interference or head impact exposure. KDT performance was not impacted by a noisy environment, supporting its sideline utility for screening more severe forms of injury.

## Introduction

The past decade has seen a significant surge in national awareness regarding the neurological risks associated with contact sports, such as American football. Over one million college and high school athletes engage in tackle football each year,^1,2^ through which they can incur several hundreds of head impacts per season.^3,4^ While these head impacts, often referred to as subconcussive impacts, do not result in concussive symptomatology, they may influence the trajectory of brain aging.^5–7^

Although subconcussive head impacts affect various brain processes and structures, ocular motor function has garnered attention among practitioners and clinician researchers due to the relative ease of evaluation procedure and functional embeddedness of the underlying neuronal circuitry.^8^ Ocular motor and neuro-ophthalmological processing involve nearly half of the brain and incorporate a diverse set of both cortical and subcortical structures whose anatomical locations render them vulnerable to the kinds of shear injury presented by subconcussive head impact.^9,10^ Thus, deficits in ocular motor function can reflect the subtle alterations in neuronal processing. For example, previous studies have demonstrated a consistent increase (worsening) in the near point of convergence (NPC) in both controlled models of subconcussive head impact and throughout the course of a football season.^11–13^ However, the complexity of neuro-ophthalmological circuitry necessitates more comprehensive tools employed in a longitudinal fashion to realize an association between head impact exposure and neuro-ophthalmological function.

The King–Devick test (KDT) was introduced in 2021 as a rapid clinical assessment tool for concussion^14,15^ and has shown promise in detecting oculomotor dysfunction related to brain injury.^16^ The KDT involves reading aloud a series of numbers displayed on a computer screen quickly and thus integrates smooth pursuit, language, and attention.^14,15^ Our recent randomized controlled trial using a controlled heading model suggests that acute subconcussive head impacts may blunt one’s ability to adapt and improve KDT performance.^17^ However, a “learning effect” has been documented after multiple KDT administrations^18,44^ posing the question of whether the conventional KDT procedure is sensitive to gauge the neuro-ophthalmological integrity after recurring exposure to subconcussive head impacts across an athletic season.

To overcome this limitation and boost the discriminatory capabilities of the KDT, in the present prospective, multisite, longitudinal study over two seasons of high school football, we incorporated sensory interference in the form of constant, diffuse auditory stimulation during KDT administration. Auditory interference has been shown to reduce cognitive performance in visual tasks through the moderation of cognitive load.^19^ The proposed mechanism for such phenomena involves thalamic processing, as the thalamus is the major structure for relaying and gating sensory afferents to the cortical regions.^20^ In support of this model, Tomasi et al.^21^ demonstrated that auditory interference reduced activity in the anterior thalamus and parietal cortex in male subjects during a visual tracking task. We hypothesized that when tested in a conventional testing method, KDT speed would remain plateaued during a season without showing a learning effect due to amounting head impact exposure. Furthermore, auditory interference during KDT administration would significantly worsen KDT speed over time. We also hypothesized that the degree of KDT worsening would be associated with head impact exposure.

## Methods

### Participants

**FIG. 1.**
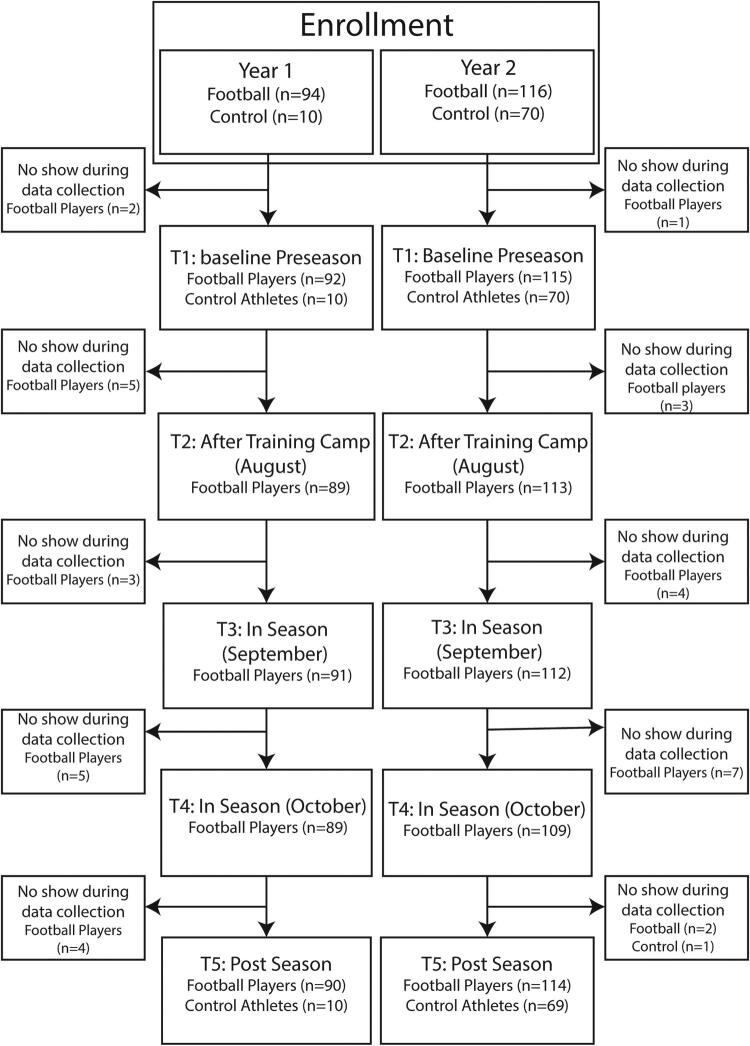
Study flow chart.

This multisite cohort study included 210 male high school football players (year 1, *n* = 94; year 2, *n* = 116) and 80 control non-contact athletes (year 1, *n* = 10; year 2, *n* = 70) from five high schools in the Midwest. The study was conducted during the 2021 and 2022 football seasons (Fig. 1). Inclusion criteria for both years consisted of being a current member of the high school football team or of the cross-country, tennis, or swimming teams for non-contact control athletes. The Indiana University Institutional Review Board approved the study protocol, and all participants and their legal guardians provided informed consent online. This study followed the Strengthening the Reporting of Observational Studies in Epidemiology (STROBE) reporting guideline (Supplementary Data 2).

### Study procedures

For both years, data collection took place during preseason in July (T1 [baseline]), during the football season in August (T2), September (T3), and October (T4), and postseason in November or December (T5). For the control athletes, data collection took place twice: T1 (baseline) in August and T5 (follow-up) in January. Study design is depicted in Supplementary Figure S1. KDT data collection for both football and control athletes was conducted in large group settings at individual schools. The year 1 cohort performed a traditional KDT, whereas the year 2 cohort performed KDT with auditory interference (see section below). During the preseason data collection, demographic information was collected from all participants, and all football players were fitted with an Impact Monitor Mouthguard (Prevent Biometrics, Inc.) to measure head impact kinematics during practices and games. For an exploratory analysis, football players in both years were categorized into three groups: low-, medium-, and high-impact, based on their head impact data.

### Head impact measurement

The impact monitor mouthguard manufactured by Prevent Biometrics was used to provide 6-degree-of-freedom spatial and temporal estimates of linear and rotational head accelerations during impact. The mouthguard uses a triaxial accelerometer (ADXL372, Analog Devices) with a maximum of 200 g per axis to detect linear acceleration. For rotational kinematics, a triaxial rotational rate gyroscope (BMG250, Bosch) was used. The mouthguard’s data acquisition system includes kinematic sensors with sufficient range and bandwidth to estimate skull motion during impact, on-board firmware for data transform from teeth to head center of gravity, nonvolatile flash memory for storage, wireless rechargeable battery, and wireless data offload using Bluetooth low energy. Accelerometer and gyroscope data were low-pass filtered at 3.2 kHz. When an axis of acceleration exceeds a preset threshold of 10 g, a standard hit duration of 50 msec of all impact data were transmitted.^22,23^ In the current study, head impacts with peak linear accelerations (PLA) > 10 g were included to distinguish kinematic events, such as jumping and running, from head impacts.^24^ Cumulative frequency, PLA, and peak rotational acceleration (PRA) over the course of a season were used in our analysis.

To validate the head impact data, we conducted a film analysis. The primary placement of the camera was at the press box. The mouthguard data were time-synchronized with film data. Randomly stratified head impact data of 1,785 head impacts were analyzed. An impact could be either to a participant’s body or head, inducing acceleration to the head. Impacts were categorized as either a true positive or false positive impact. Positive predictive values or precision were computed by dividing true positives by the sum of true positives and false positives. Our analyses resulted in 1,670 out of 1,785 impacts (94%) aligning between mouthguard data and film analysis. Of the 6% of impacts that did not agree, 4% were from games and 2% from practices.^25^

### KDT assessment

Using a tablet device, the KDT examines neuro-ophthalmological function by performing a total of 145 saccades while rapidly reading numbers aloud. The participants were given one trial of demonstration cards for practice, followed by three different test cards. The tablet recorded the total amount of time it took participants to complete all three cards.^14,15^ The year 2 cohort wore noise-canceling headphones, and throughout the KDT assessment, constant traffic noise sounds at a high volume were played into the headphones to induce auditory interference. The testers stood behind the participants and recorded any errors made during the testing. Given that the KDT error is a subjective measure, and many research staff were involved in data collection, we set the KDT speed as our primary outcome measure.

### Statistical analysis

Differences in demographic variables between year 1 and 2 cohorts were assessed by independent sample t-tests and chi-square tests. A series of mixed-effects regression models (MRMs) was used to examine our hypotheses, with KDT time as the primary outcome for all models. The first set of MRMs assessed time-course changes in traditional KDT (for year 1) and auditory interference KDT performance (for year 2) by evaluating time effects, group effects, and group-by-time interactions. The model was adjusted by covariates, including age, years of tackle football experience, and number of previous concussions. To account for multiple time points (*n* = 5), the level of significance was corrected to *p* < 0.01.

Second, we implemented quantile-based binning^26^ based on the sum of head impact count during a season and categorized participants in each cohort into three groups (high, medium, low impact). Given that the sum of head impact frequency and head impact kinematics were highly correlated (PLA r = 0.993; PRA r = 0.964), we used the head impact frequency for this cluster analysis. Please see Supplementary Data S1. We then used an MRM to determine whether KDT performance throughout the season differed between head impact groups in each year.

Lastly, we employed a similar MRM for the control group to evaluate the degrees of changes in KDT performance between preseason and postseason in years 1 and 2. Due to differences in the frequency of data collection between football (*n* = 5 per season) and control groups (*n* = 2 per season) and unbalanced sample sizes in the control group in year 1 (*n* = 10) and year 2 (*n* = 70), we refrained from conducting group analyses between the football and control groups. All analyses were conducted using R, version 4.2.1 (R Project for Statistical Computing) with the nlme package.

## Results

### Demographics

For year 1, 94 high school football players (mean [SD] age, 15.8 [1.1] years) were enrolled (Table 1). Year 2 of the study included 116 high school football players (age, 15.5 [1.3]). All athletes were males and predominantly White (84.5–88.3%). There were no significant differences in any demographic variables between years 1 and 2. These football players were further categorized into high, medium, and low impact groups, as follows: year 1 high *n* = 32, medium *n* = 31, low *n* = 31; year 2 high *n* = 39, medium *n* = 38, low *n* = 39 (Supplementary Table 1). Ten and 70 control athletes were enrolled in the year 1 and 2 of the study, respectively (Supplementary Table 2), with a similar demographic background to football players.

**Table 1.** Group Demographics and Head Impact Kinematics in Football Players

Group	Year 1	Year 2	p-value
*n*	94	116	—
Sex (%)	94 M (100%)	116 M (100%)	—
Age, y	15.8 (1.1)	15.5 (1.3)	0.062
BMI, kg/m^2^	26.1 (5.4)	26.5 (5.6)	0.586
No. of previous concussion			0.199
0, *n* (%)	79 (84.0)	90 (77.5)	—
1, *n* (%)	11 (11.7)	21 (18.1)	—
2, *n* (%)	4 (4.3)	5 (4.4)	—
Tackle football experience, y	5.1 (2.9)	4.8 (3.4)	0.551
Race, *n* (%)			0.301
White	83 (88.3)	98 (84.5)	—
Black/African American	9 (9.6)	11 (9.5)	—
Asian	0 (0)	6 (5.2)	—
American Indian or Alaska Native	0 (0)	1 (0.8)	—
Native Hawaiian or Pacific Islander	0 (0)	0 (0)	—
Multiracial	2 (2.1)	0 (0)	—
Ethnicity, *n* (%)			1.00
Not Latino/Hispanic	88 (93.6)	110 (94.8)	—
Latino/Hispanic	6 (6.4)	6 (5.2)	—
Average-impact kinematics for season (SD)			
Cumulative impact count	102 (113)	92.6 (95.0)	0.585
Cumulative PLA, *g*	1641.6 (1856.2)	1564.1 (1605.1)	0.751
Cumulative PRA, krad/s^2^	32.5 (39.4)	105.08 (116.3)	0.536

Note: BMI, body mass index; SD, standard deviation; PLA, peak linear acceleration; PRA, peak rotational acceleration.

### Traditional (year 1) vs. auditory interference (year 2) KDT performance

**FIG. 2.**
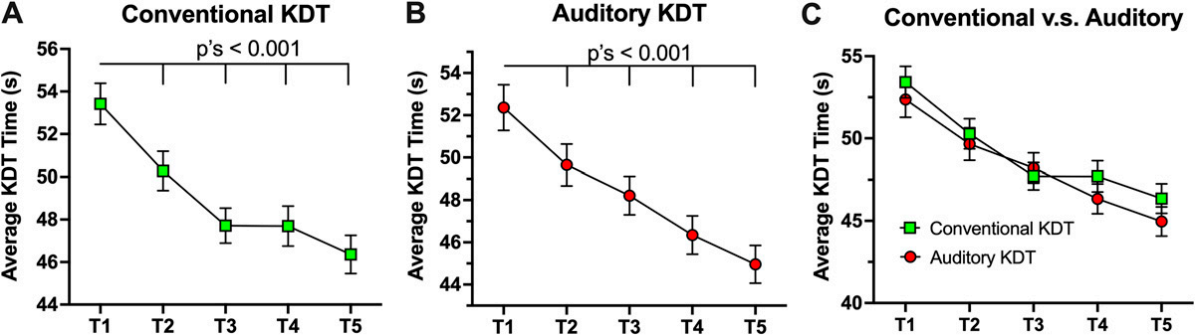
Time-course changes in KDT speed. Significant decrease (fastened) in KDT speed was observed over the course of a season in conventional KDT **(A)** and auditory KDT **(B)**. The combined figure is presented in a panel C. KDT, King–Devick test.

When players performed traditional KDT, they continued to demonstrate significant improvements in their KDT speed, as illustrated by statistically significant time effects (β = −1.7, SE = 0.12, *p* < 0.01: Fig. 2A). On average (SD), KDT times were 53.42 (9.3) sec at preseason and 46.35 (8.5) sec at postseason. See Supplementary Table S3 for the statistical output and Supplementary Table S4 for average KDT times at each time point. Similarly, the football players performing the KDT assessment with auditory interference in year 2 demonstrated significant improvements in KDT time (β = −1.7, SE = 0.11, *p* < 0.001: Fig. 2B). On average (SD), the KDT times for the year 2 football players were 52.3 (11.5) sec at preseason and 45.07 (9.5) sec at postseason. The degrees of improvement in KDT performance were very similar between traditional KDT and auditory interference KDT, as illustrated by no significant KDT group by time interaction (β = −0.08, SE = 0.17, *p* = 0.65) (Fig. 2C).

### The role of head impact exposure in KDT performance

**FIG. 3.**
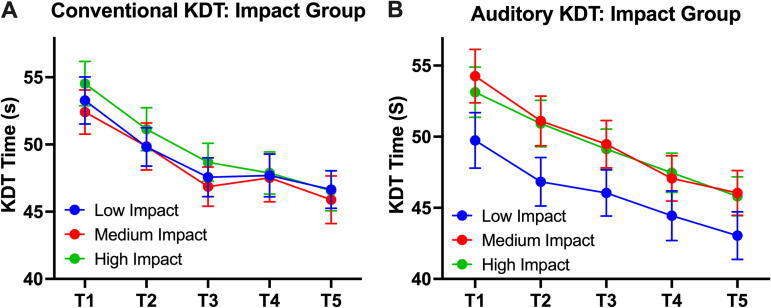
Time-course changes in KDT speed per head impact group. Football players were clustered into either high-, medium-, or low-impact group. All groups demonstrated similar degrees of improvement in conventional KDT and auditory KDT. KDT, King–Devick test.

The football players in each year were then clustered into three groups: low, medium, and high impacts. In year 1, all groups improved KDT times to a similar degree with no significant difference in KDT times based on the head impact groups (β = 0.21, SE = 0.2, *p* = 0.84: Fig. 3A), with no significant group-by-time interaction (β_year 1_ = −0.12, SE = 0.15, *p* = 0.41) (Table 2). These cluster analysis results were replicated in year 2 even under conditions of auditory interference. There was no statistically significant difference between the head impact groups and their KDT times throughout the course of the year 2 season (β = 1.7, SE = 0.98, *p* = 0.09), (Fig. 3B) which is also illustrated by no significant group-by-time interaction (β_year 2_ = −0.15, SE = 0.14, *p* = 0.26).

**Table 2.** Group Differences in Outcome (KDT) Variables at Each Timepoint. Preseason (T1) as a Reference Time Point

		T2 post-camp (August)	T3 in-season (September)	T4 in-Season (October)	T5 postseason (November)
Average					
	Year 1	−3.4 (0.5)^***^	−5.4 (0.5)^***^	−5.9 (0.5)^***^	−7.2 (0.5)^***^
	Year 2	−2.7 (0.5)^***^	−4.1 (0.5)^***^	−5.9 (0.5)^***^	−7.3 (0.5)^***^
Year 1					
	Low Impact	−2.9 (0.8)^***^	−5.3 (0.8)^***^	−5.7 (0.8)^***^	−6.7 (0.8)^***^
	Medium Impact	−2.8 (0.8)^***^	−5.1 (0.8)^***^	−5.3 (0.8)^***^	−6.9 (0.8)^***^
	High Impact	−3.4 (0.8)^***^	−5.8 (0.8)^***^	−6.6 (0.8)^***^	−7.9 (0.8)^***^
Year 2					
		Low Impact	−3.0 (0.9)^***^	−3.5 (0.9)^***^	−5.2 (0.9)^***^	−6.7 (0.9)^***^
	Medium Impact	−3.2 (0.8)^***^	−4.8 (0.8)^***^	−6.9 (0.8)^***^	−8.2 (0.8)^***^	
	High Impact	−2.2 (0.9)^*^	−4.1 (0.9)^***^	−5.9 (0.9)^***^	−7.3 (0.9)^***^	

Note: Values are expressed as difference (95% confidence interval). KDT, King–Devick Test.

^*^ *p* < 0.05.

^***^ *p* < 0.001.

### KDT performance in control athletes

Similar to the football players, the control athletes exhibited a significant decrease in KDT time from preseason to postseason (β = −1.6, SE = 1.9, *p* < 0.001) (Supplementary Fig. 2A). On average (SD), KDT speed for the control athletes in year 1 was 46.57 (6.1) sec at preseason and 40.07 (5.3) sec at postseason. The identical pattern was observed in control athletes in year 2 while performing KDT with auditory interference [preseason: 51.68 (11.6) sec; postseason 48.98 (9.9) sec: β = −0.7, SE = 0.2, *p* = 0.001] (Supplementary Fig. 2B).

## Discussion

The novelty of this study involved a repeated-measures analysis of the relationship between repetitive subconcussive head impacts and neuro-ophthalmological function in high school football players over the course of a full season. In addition, we applied an innovative concept using auditory interference to enhance the diagnostic sensitivity of the KDT, introducing a unique multisensory modality for the evaluation of neuro-ophthalmological function. Contrary to our hypotheses, (1) we observed continuous improvement of KDT performance throughout the course of the football season, (2) nor did the introduction of auditory interference serve to potentiate any subconcussive damage that may have gone undetected under standard KDT testing conditions. (3) Our cluster analysis, which grouped players according to high, medium, and low head impact groups, yielded no significant differences in KDT progression between groups. Collectively, our data suggest that there is no clear relationship between subconcussive head impact and neuro-ophthalmological dysfunction in a longitudinal design and that noise introduced during the KDT had no impact on one’s ability to perform and improve their KDT performance.

The inspiration for auditory interference during KDT administration was derived from well-established dual-task evaluative paradigms aimed at identifying subtle indications of brain damage. These diagnostic models have been limited mostly to the realm of neuromuscular dysfunction, often combining a cognitive task with a motor task to potentiate any impact-induced deficits in balance or locomotion.^27–29^ The success of models, such as these, relies upon the considerable structural overlap in the neural execution of both cognitive and motor tasks.^30^ Correspondingly, we sought to take advantage of shared pathways for the integration of both auditory and visual stimuli, particularly targeting the sensory gating role of the thalamus.^20,31,32^ Sound-induced deficits in visual task performance have been demonstrated repeatedly, and imaging results have revealed alterations in thalamic activity when engaged in a visual tracking task under conditions of auditory interference.^19,21,33^ However, conflicting evidence exists. Several studies suggest the independent nature of auditory and visual/ocular-motor functions,^34,35^ such that auditory stimulation may have no effect on visual performance. Furthermore, it has also been demonstrated that auditory stimulation may actually facilitate visual performance in some tasks.^21,33^ A more recent analysis of multisensory research has generated a model of *task dependence* for sensory resource allocation, which posits that object-based visual tasks (e.g., identifying a stimulus) are processed independently from auditory function, whereas considerable overlap across attentional resources for spatial visual tasks (e.g., determining stimulus location) and auditory processing.^36^ Given the spatial nature of the KDT in addition to its object-based aspect, we predicted that our model of auditory interference would in fact alter participants’ efficiency in performing the KDT and that this added difficulty would lessen the brain’s ability to compensate for subconcussive damage. Our aspiration for diagnostic enhancement ultimately proved unsuccessful, likely due to the complexity of the relationship between these sensory pathways, which is also reflected by the contradictory nature of previous research dedicated to the topic.

The outcome of this study was inconsistent with previous findings relating to neuro-ophthalmological dysfunction with repeated subconcussive head impact exposure. Our previous study which utilized soccer-heading to employ a controlled model of subconcussive head impact found a significant impact-induced dampening of the KDT learning curve.^17^ The primary reasons for this discrepancy may be due to the lengths of the longitudinal design and the interval between impact exposure and KDT assessment. In the controlled model, participants performed the KDT immediately, 2 h, and 24 h after 10 head impacts. In the current study, participants were administered the KDT before games at four separate time points throughout the season. Given that the teams incorporate non-contact practice a day before games, the KDT performance is likely free of acute head impact effects. This suggests that the KDT performance may be influenced by acute exposure to head impacts, but the impairment is transient and normalized quickly; hence, our KDT data did not reflect the cumulative effects of subconcussive head impacts.

Time plays a significant role in the healing of all forms of mild traumatic brain injury,^37^ and the time interval between successive head impacts has been shown to be a strong determinant of total inflicted damage.^38,39^ While our data do not support the notion of any effect of subconcussive head impacts from a season of tackle football on neuro-ophthalmological processing, it is unlikely that function is entirely retained, given the amount of previous data, which suggest otherwise.^11–13^ Instead, our findings can be seen as suggestive of a relatively quick oculomotor recovery or accommodation window following impact exposure. Several studies have found a temporal window of brain vulnerability after successive mild traumatic brain injuries lasting up to 72 h after exposure;^38–42^ however, the results of our study suggest that specifically within the realm of subconcussive head impacts and neuro-ophthalmological processing, certain functions may be recovered within 24 h. Moreover, given the KDT’s wide scope of neuronal evaluation, which integrates attention, language, and concentration,^43^ it is plausible that cognitive accommodation plays an important role in maintaining KDT performance despite incurring subconcussive head impacts.

### Clinical relevance

While the KDT has shown promise for the role of detecting concussion, our data compiled over two years suggest a lack of sensitivity for subconcussive damage sustained over a season of tackle football. Participants showed resilience to both traditional and auditory interference KDT testing modalities. Unlike isolated oculomotor functions, such as the NPC,^11^ KDT’s inclusion of diverse cognitive functions points to the role of cognitive accommodation. Therefore, the KDT may be inadequate for the evaluation of subconcussive brain damage. However, an important message has been raised from this study, in that the KDT performance does not alter from the loud noisy environment, which is useful information for screening for concussive injury in a noisy, sideline setting. Follow-up research incorporating multisensory stimuli is needed to calibrate the diagnostic accuracy of various clinical neurological assessments.

### Limitations

There are several limitations recognized in this study and the findings should be interpreted with those in mind. Most notably, the sample for this study was gathered from a predominantly White and non-Hispanic/Latino population in the American Midwest. This study would benefit from being reproduced with more racial and ethnic diversity. Another limitation of the study is that the entirety of the sample is male since tackle football is dominated by a male population. Therefore, the findings of this study cannot be generalized to a female population. Finally, the difference in sample size in control athletes from year 1 to year 2 was a limitation. This difference was due to recruitment for the first year of the study taking place during the height of the COVID-19 pandemic; thus, the sample size for control athletes was 10, as opposed to 70 controls in year 2.

## Conclusion

The current study aimed to examine the impact of auditory interference on the relationship between repetitive subconcussive head impacts and neuro-ophthalmological processing in adolescent football players. Our data suggest that KDT performance improves during a season regardless of auditory interference or the number of head impacts sustained. Therefore, KDT may not be a suitable tool to detect cumulative subconcussive head impacts in adolescent football players. Yet, KDT performance was not impacted by a noisy environment, which is useful information for concussion screening in the sideline setting.

### Transparency, rigor, and reproducibility statement

This cohort study included high school football players and non-contact control athletes. The year 1 cohort was tested with a conventional KDT, whereas the year 2 cohort was tested with a KDT with auditory interference. A sample size of 80 in each year was planned to yield 80% power to detect statistically significant KDT condition effects with a *p*-value < 0.05. Ninety-four high school football players were screened, and all participants were enrolled in year 1, and in year 2 of the study, 116 high school football players were screened and enrolled. For the control group, 10 and 70 players were screened and enrolled in year 1 and 2, respectively. Participants were blinded regarding any study aims and final outcome and will be referred to the publication when it becomes available. Statistical analyses were performed by the team member blinded to relevant characteristics of the participants. All equipment and software used to perform analyses are widely available from commercial sources. De-identified data from this study and analytic code are available upon reasonable request to the corresponding author (KK).

## Supplementary Material

Supplementary Figure S1

Supplementary Figure S2

Supplementary Table S1

Supplementary Table S2

Supplementary Table S3

Supplementary Table S4

Supplementary Data S1

Supplementary Data S2

## Abbreviations Used

KDT: King-Devick Test
MRMs: Mixed-effects regression models
NPC: Near point of convergence
PLA: Peak linear acceleration
PRA: Peak rotational acceleration
SD: Standard deviation
STROBE: Strengthening the Reporting of Observational Studies in Epidemiology
