# Supplementary material for: Association of Auditory Interference and Ocular-Motor Response with Subconcussive Head Impacts in Adolescent Football Players
Source: Neurotrauma Rep. 2024 May 31;5(1):512–21. doi: 10.1089/neur.2023.0125 (PMC11295109; doi:10.1089/neur.2023.0125)

**Supplemental Documents**


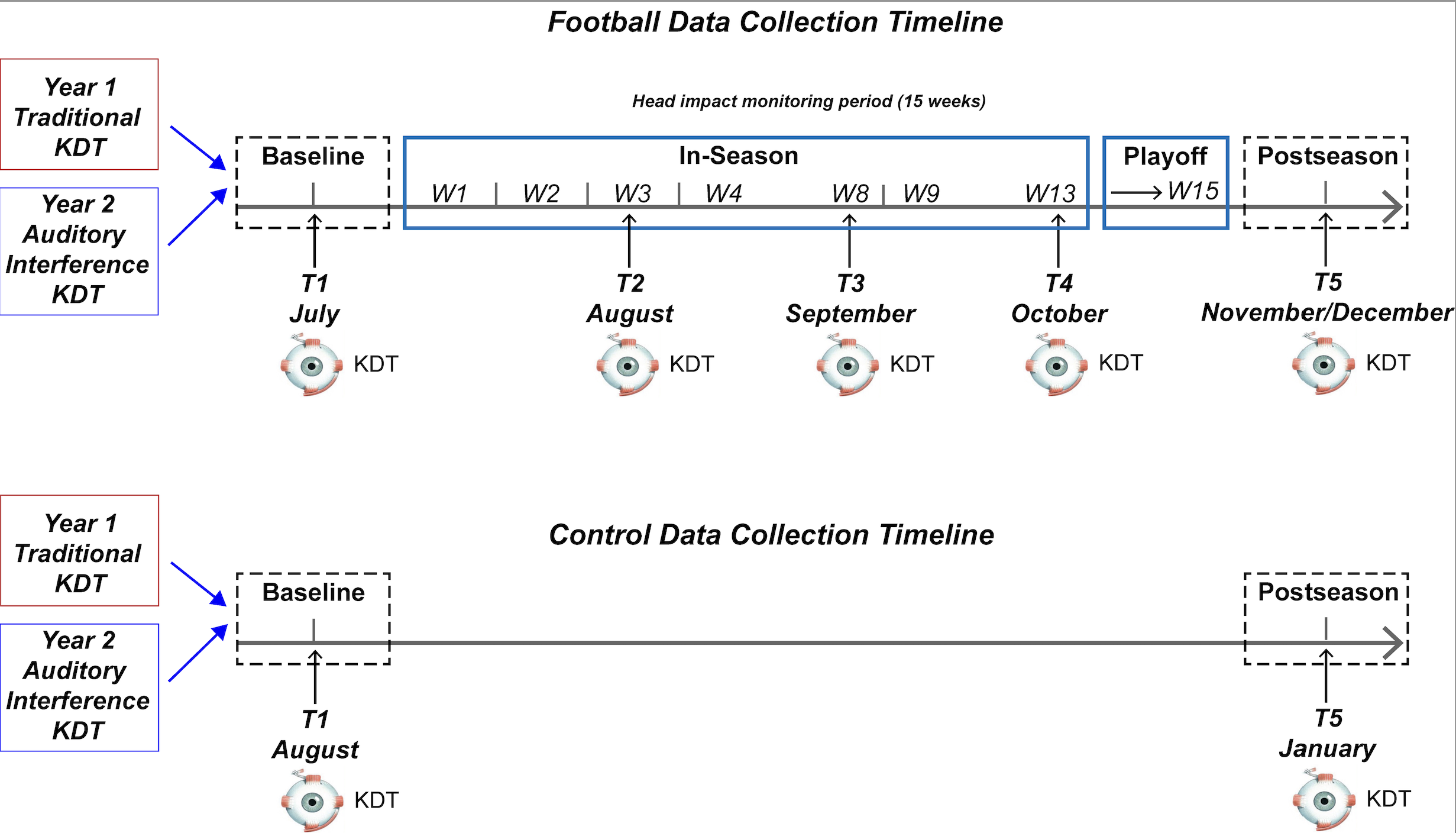


**sFigure 1: Study design schematics**


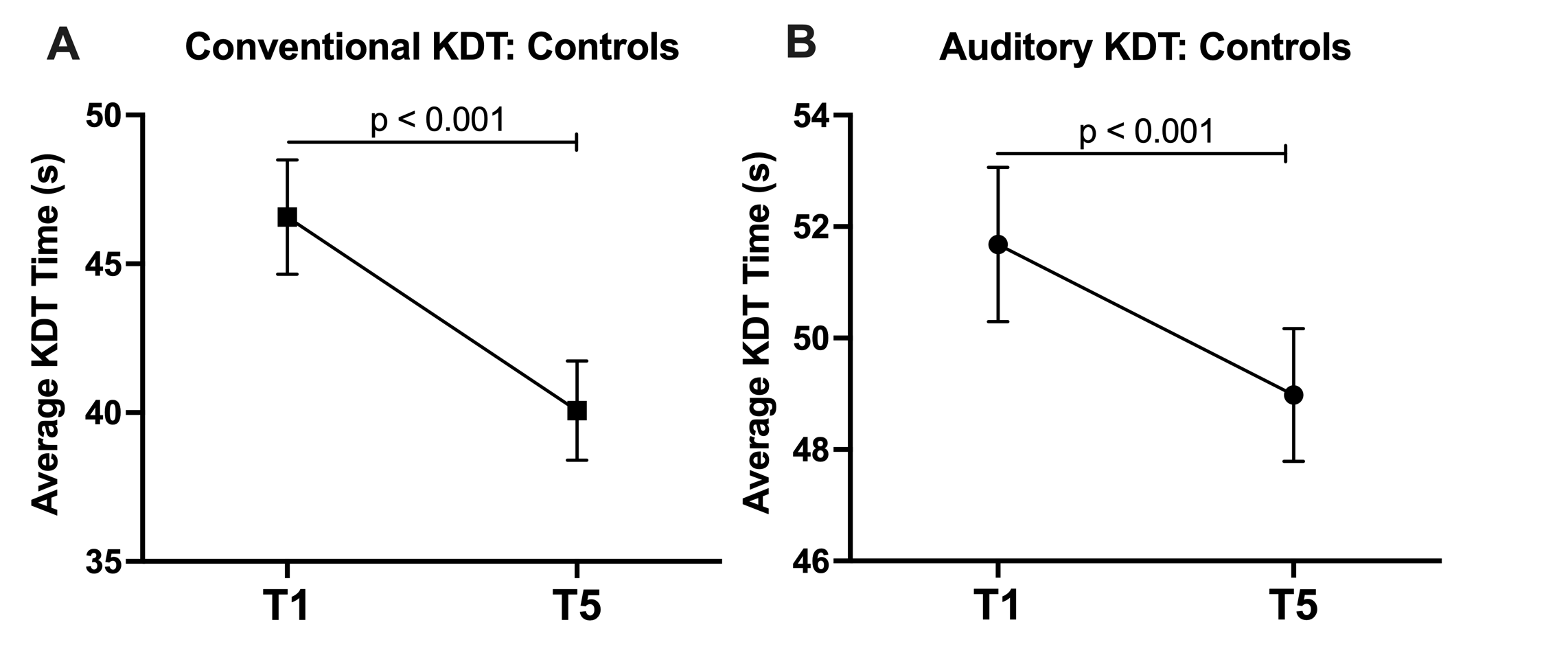


**sFigure 2: KDT changes at post-season compared to pre-season baseline**. Significant improvement (fastened) in KDT speed was observed over the course of a season in conventional KDT (A) and auditory KDT (B).

| **sTable 1. Head impact cluster demographics and head impact kinematics** | | | | | | |
| --- | --- | --- | --- | --- | --- | --- |
| **Year** | **Year 1** | | | **Year 2** | | |
| **Impact Cluster** | **High** | **Medium** | **Low** | **High** | **Medium** | **Low** |
| n | 32 | 31 | 31 | 39 | 38 | 39 |
| Sex (%) | 32 M (100%) | 31 M  (100%) | 31 M (100%) | 39 M (100%) | 38 M (100%) | 39 M (100%) |
| Age, y | 15.9 (1.2) | 15.8 (0.9) | 15.7 (1.2) | 15.7 (1.3) | 15.3 (1.2) | 15.5 (1.4) |
| BMI, kg/m^2^ | 27.5 (4.7) | 25.8 (6.32) | 25 (4.8) | 26.6 (4.7) | 26.8 (6.2) | 26.2 (5.9) |
| No. of previous concussion |  |  |  |  |  |  |
| 0, *n* (%) | 28 (87.5) | 25 (80.6) | 26 (83.9) | 31 (79.5) | 30 (78.9) | 29 (74.4) |
| 1, *n* (%) | 3 (9.4) | 4 (12.9) | 4 (12.9) | 6 (15.4) | 8 (21.1) | 7 (17.9) |
| 2, *n* (%) | 1 (3.1) | 2 (6.5) | 1 (3.2) | 2 (5.1) | 0 (0) | 3 (7.7) |
| Tackle football experience, y | 6 (2.9) | 4.03 (2.4) | 5.19 (3.0) | 5.2 (3.04) | 4.3 (3.6) | 4.9 (3.5) |
| Race, *n* (%) |  |  |  |  |  |  |
| White | 28 (87.5) | 27 (87.1) | 28 (90.3) | 33 (84.6) | 32 (84.2) | 33 (84.6) |
| Black/African American | 2 (6.25) | 4 (12.9) | 3 (9.7) | 4 (10.3) | 4 (10.5) | 3 (7.7) |
| Asian | 0 (0) | 0 (0) | 0 (0) | 2 (5.1) | 2 (5.3) | 2 (5.1) |
| American Indian or Alaska Native | 0 (0) | 0 (0) | 0 (0) | 0 (0) | 0 (0) | 1 (2.6) |
| Native Hawaiian or Pacific Islander | 0 (0) | 0 (0) | 0 (0) | 0 (0) | 0 (0) | 0 (0) |
| Multiracial | 2 (6.25) | 0 (0) | 0 (0) | 0 (0) | 0 (0) | 0 (0) |
| Ethnicity, *n* (%) |  |  |  |  |  |  |
| Not Latino/Hispanic | 29 (93.5) | 29 (93.5) | 30 (96.8) | 37 (94.9) | 36 (94.7) | 37 (94.9) |
| Latino/Hispanic | 3 (9.4) | 2 (6.5) | 1 (3.2) | 2 (5.1) | 2 (5.3) | 2 (5.1) |
| Average impact kinematics for season (SD) |  |  |  |  |  |  |
| Cumulative impact count | 227.0  (109.0) | 62.7  (19.8) | 14.6  (10.0) | 192.6 (102.8) | 66.5  (16.6) | 19.7  (13.6) |
| Cumulative PLA, *g* | 3710.5  (1935.8) | 989.7  (318.6) | 224.1  (158.5) | 3241.5  (1763.8) | 1064.1  (391.4) | 391  (267.7) |
| Cumulative PRA, krad/s^2^ | 262.3  (167.5) | 70.8  (29.3) | 17.0  (11.3) | 216.1  (145.6) | 80.3  (26.4) | 20.3  (14.7) |
| Note: BMI, body mass index. SD, Standard Deviation. PLA, peak linear acceleration. PRA, peak rotational acceleration. | | | | | | |

| **sTable 2. Control & football demographics** | | | | | | |
| --- | --- | --- | --- | --- | --- | --- |
| **Year** | **Year 1** | | | **Year 2** | | |
| **Group** | **Contact** | **Control** | **P-value** | **Contact** | **Control** | **P-value** |
| **n** | 94 | 10 | - | 116 | 70 | - |
| **Sex (%)** | 94 (100) | 10 (100) | - | 116 (100) | 70 (100) | - |
| **Age, (SD)** | 15.8 (1.1) | 15.7 (1.2) | 0.8 | 15.5 (1.3) | 15.9 (1.2) | 0.03 * |
| **BMI, kg/m^2^** | 26.1 (5.4) | 19.6 (1.4) | <0.001** | 26.5 (5.6) | 21.7 (3.7) | <0.001** |
| **No. of previous concussion** |  |  |  |  |  |  |
| 0, *n* (%) | 79 (84.0) | 9 (90) | - | 90 (77.5) | 63 (90) | - |
| 1, *n* (%) | 11 (11.7) | 1 (10) | - | 21 (18.1) | 6 (8.6) | - |
| 2, *n* (%) | 4 (4.3) | 0 (0) | - | 5 (4.4) | 1 (1.4) | - |
| **Race, *n* (%)** |  |  | 0.23 |  |  | 0.17 |
| White | 83 (88.3) | 9 (90) | - | 98 (84.5) | 63 (90) | - |
| Black/African American | 9 (9.6) | 0 (0) | - | 11 (9.5) | 1 (1.4) | - |
| Asian | 0 (0) | 0 (0) | - | 6 (5.2) | 5 (7.2) | - |
| American Indian or Alaska Native | 0 (0) | 0 (0) | - | 1 (0.8) | 0 (0) | - |
| Native Hawaiian or Pacific Islander | 0 (0) | 0 (0) | - | 0 (0) | 1 (1.4) | - |
| Multiracial | 2 (2.1) | 1 (10) | - | 0 (0) | 0 (0) | - |
| **Ethnicity, *n* (%)** |  |  | 0.91 |  |  | 1 |
| Not Latino/Hispanic | 88 (93.6) | 10 (100) | - | 110 (94.8) | 67 (95.7) | - |
| Latino/Hispanic | 6 (6.4) | 0 (0) | - | 6 (5.2) | 3 (4.3) | - |
| **Sports played, *n (%)*** |  |  |  |  |  |  |
| Football | 94 (100) | 0 (0) | - | 116 (100) | 0 (0) | - |
| Cross-country | 0 (0) | 8 (80) | - | 0 (0) | 24 (34.2) | - |
| Tennis | 0 (0) | 2 (20) | - | 0 (0) | 23 (32.9) | - |
| Swimming | 0 (0) | 0 (0) | - | 0 (0) | 23 (32.9) | - |
| **Note:** BMI, body mass index. SD, Standard Deviation. | | | | | | |

| **sTable 3: Differences in outcome variables at each timepoint as a function of year. Pre-Season (T1) as a reference time point.** | | | | | |
| --- | --- | --- | --- | --- | --- |
|  |  | T2 Post-camp  (August) | T3 In-season  (September) | T4 In-Season  (October) | T5 Postseason  (November) |
| KDT  in seconds | Year 1 | -3.4 (0.5)*** | -5.4 (0.5)*** | -5.9 (0.5)*** | -7.2 (0.5)*** |
|  | Year 2 | -2.7 (0.5)*** | -4.1 (0.5) *** | -5.9 (0.5) *** | -7.3 (0.5) *** |
| Note: Values are expressed as difference (95% confidence interval). The unit for KDT output is seconds. KDT, King Devick Test. *p<0.05, **p<0.01, ***p<0.001. | | | | | |

| **sTable 4: Average KDT Times in seconds at Each Timepoint for Football Players** | | | | | | |
| --- | --- | --- | --- | --- | --- | --- |
|  |  | T1 – Pre-Season  (July) | T2 Post-camp  (August) | T3 In-season  (September) | T4 In-Season  (October) | T5 Postseason  (November) |
| Average | Year 1 | 53.4 (9.3) | 49.9 (9.8) | 47.7 (7.9) | 47.7 (8.9) | 46.3 (8.5) |
|  | Year 2 | 52.4 (11.6) | 49.8 (10.5) | 48.3 (9.7) | 46.5 (9.5) | 45.1 (9.6) |
| Year 1 | Low Impact | 53.5 (9.4) | 49.4 (7.6) | 47.6 (7.7) | 47.7 (8.1) | 46.5 (7.4) |
|  | Medium Impact | 52.5 (9.2) | 49.1 (12.9) | 47 (8.2) | 47.9 (10.2) | 46.1 (10.1) |
|  | High Impact | 54.1 (9.4) | 51.2 (8.6) | 48.4 (7.9) | 47.5 (8.6) | 46.6 (8.1) |
| Year 2 | Low Impact | 49.7 (12.2) | 46.8 (10.4) | 46 (10) | 44.4 (10.5) | 43.2 (10.4) |
|  | Medium Impact | 54 (11.5) | 51.1 (10.8) | 49.5 (10.3) | 47.1 (9.6) | 46.1 (9.7) |
|  | High Impact | 53.4 (11) | 51.3 (10.1) | 49.4 (8.5) | 47.8 (8.4) | 46.1 (8.6) |
| Note: Values are expressed as average (standard deviation). KDT, King Devick Test. | | | | | | |

Supplementary Statistical Methods

Quantile-based binning is a statistical method that is used to divide a continuous variable into categories or ‘bins’ based on specific quantiles of the variables’ distribution. This method is commonly used when one wants to create groups with approximately equal sizes or if one wants to divide the data into meaningful segments based on percentiles.

The first step of quantile-based binning is calculating the quantiles of the continuous variable. For our analysis, our continuous variable was frequency of head impacts that was measured with the vector-based mouthguard. We knew we wanted our impacts divided into three approximately equal bins. Since the quantiles are used as breakpoints to define the boundaries of the bins, our cutoffs were set a 1, 1/3, 2/3, and 1. Once breakpoints are defined, each data point is assigned to a bin based on its value, or frequency of head impacts. The three bins were labelled as “Low”, “Medium”, and “High” respectively to make the data more easily interpretable. Please see below for the RStudio code that was used.


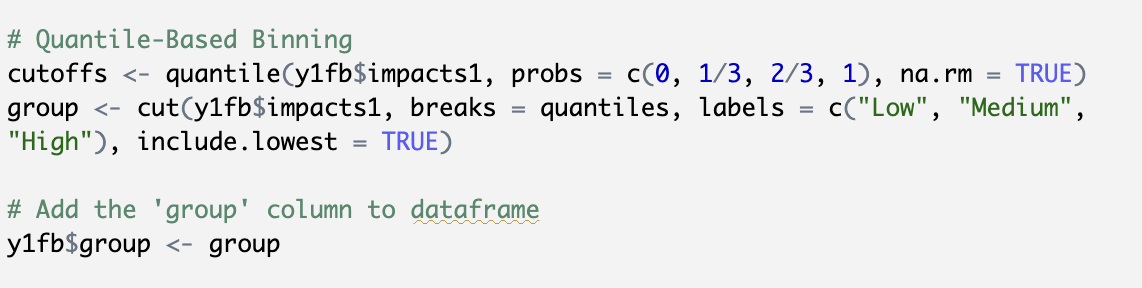

Supplement: Supplementary Figure S1 [file neur.2023.0125_supplemental_documents.docx]
